# Innovative 3D-printed internal structure in silicone partial-hand prostheses: Enhancing function and mechanical performance

**DOI:** 10.33137/cpoj.v9i1.47092

**Published:** 2026-06-20

**Authors:** M. Mohammad Zadeh, M. Jalali, A. Khaghani

**Affiliations:** Rehabilitation Research Center, Department of Orthotics and Prosthetics, School of Rehabilitation Sciences, Iran University of Medical Sciences, Tehran, Iran.

**Keywords:** Cosmetic Prosthesis, Partial Hand, Silicone, 3D Printing, Satisfaction, Amputation, Functional Ability

## Abstract

**BACKGROUND:**

Silicone cosmetic prostheses play a significant role in improving body image and reducing psychosocial issues among individuals with partial hand amputation; however, high weight and insufficient stiffness are their primary limitations.

**OBJECTIVES:**

To design a novel silicone cosmetic prosthesis with a lightweight and durable internal structure using 3D printing and to evaluate its impact on user satisfaction and functional ability scores.

**METHODOLOGY:**

This pilot semi-experimental pre–post study was conducted between February and September 2024 at a prosthetics and orthotics center in Tehran, on 7 individuals (4 males, 3 females; mean age 27.9 ± 7.6 years) with unilateral partial hand amputation. For each participant, a silicone cosmetic prosthesis with a 3D-printed internal thermoplastic polyurethane (TPU) structure was fabricated. User satisfaction was assessed using the TAPES questionnaire, and functional ability was evaluated using the DASH questionnaire. Prosthesis stiffness was measured using a force–displacement test, and fatigue resistance was assessed through a displacement-controlled cyclic loading test (25 mm) until failure. Outcomes were compared with participants’ existing conventional full-silicone prostheses.

**RESULTS:**

The results indicated that the TPU prosthesis was lighter than the full-silicone prosthesis (0.200 ± 0.05 vs. 0.29 ± 0.09 kg; p = 0.001), stiffer (81.43 ± 27.07 vs. 27.29 ± 10.83 N; p = 0.028), and more resistant to fatigue in the tested sample (36,500 vs. 13,800 cycles). The mean DASH score was lower for the TPU prosthesis (43.69 ± 11.67 vs. 53.09 ± 11.56; p = 0.012), indicating superior functional ability. Additionally, overall satisfaction (TAPES) and separately measured satisfaction with prosthesis stiffness were higher in the TPU prosthesis. Participants reported longer daily wear time with the TPU prosthesis.

**CONCLUSION:**

In this pilot sample, silicone prostheses incorporating a 3D-printed internal TPU structure demonstrated improved mechanical and functional outcomes compared to conventional full-silicone prostheses. Despite the limitation of small sample size, these preliminary findings highlight the potential value of integrating lightweight and durable materials through 3D printing in the design of cosmetic partial-hand prostheses. Future studies with larger sample sizes are necessary to confirm and generalize these results.

## INTRODUCTION

The human upper limb is a complex structure comprising neurovascular, lymphatic, muscular, and skeletal components that function in harmony to enable coordinated kinematic chain movements.^1^ The hand, as a pivotal element of this limb, possesses 19 degrees of freedom, facilitating both fine and gross motor activities, self-care, social interactions, and self-expression.^1–3^

Numerous factors, including infections, trauma, cancer, and congenital anomalies, can lead to upper limb amputation.^1^

Recent estimates indicate that approximately 2.3 million individuals in the United States are living with limb loss, including about 9.2% with upper extremity amputations. This number is projected to increase by approximately 145% by 2060.^4^ Among upper limb amputations, transcarpal amputations account for approximately 61% of cases, representing the most common level of limb loss.^5^ These injuries typically result from severe damage to the fingers, thumb, or metacarpal regions.^6^ Partial hand amputation not only causes physical disability but also exerts a profound psychosocial impact on the individual, often equated to the loss of a loved one, eliciting a similar grieving process. This form of amputation adversely affects functional ability, body image, quality of life, and life satisfaction.^7,8^

One approach to mitigating the consequences of amputation is the use of prostheses.^9^ Prostheses serve as devices to replace lost limbs, enabling individuals to partially regain prior activities.^10^ Prostheses for this level of amputation are categorized into five types: passive functional, body-powered active, externally powered active, activity-specific, and hybrid.^5^ Passive prostheses are commonly employed when aesthetics and comfort are prioritized, and they are preferred by users due to their more natural appearance and enhanced comfort, allowing continuation of professional and social life.^6,11,12^

Cosmetic prostheses, classified as passive functional devices, represent one of the oldest and most widely used types of prostheses. They are fabricated for any level of partial hand amputation to restore a natural hand appearance.^13^ These prostheses are meticulously matched in size, shape, surface details, and color to the contralateral side, appearing highly natural to observers.^14^ The natural appearance aids in restoring body image, improving mental health, and reducing unwanted attention.^15^ Despite functional limitations, cosmetic prostheses enable certain activities such as pushing, pulling, carrying objects, typing, and holding items.^3,13^ A study by Fraser demonstrated that in activities not requiring active hand movements, the performance of passive prostheses, except for pulling objects, shows no significant difference from active prostheses.^16^ Cosmetic prostheses are primarily constructed from silicone, and silicone partial hand prostheses offer a highly natural appearance with excellent conformity to body tissues.^17^

It has been proposed to incorporate a semi-rigid internal structure within the fingers to provide the necessary stiffness for specific tasks, thereby improving the individual's functional ability. This internal structure is typically made from braided stainless steel to ensure sufficient durability during repeated flexion and extension; however, increased weight and the potential for silicone failure or tearing represent key limitations.^11,14^ Studies indicate that the primary design priorities for prostheses include comfort, aesthetic properties, and functionality.^18,19^ To address these limitations, thermoplastic polyurethane (TPU), a lightweight and durable material suitable for 3D printing, can be used as the internal structural material. Incorporating a semi-rigid internal structure using lightweight materials such as thermoplastic polyurethane (TPU) may enhance mechanical performance and improve functional ability in daily activities.^20^

The present study aimed to compare a silicone cosmetic prosthesis incorporating a 3D-printed TPU internal structure with a conventional full-silicone prosthesis in terms of functional ability and user satisfaction in individuals with unilateral partial hand amputation.

It was hypothesized that the incorporation of a 3D-printed thermoplastic polyurethane (TPU) internal structure within silicone cosmetic prostheses would result in improved mechanical properties, higher user satisfaction, and enhanced functional performance compared to conventional full-silicone prostheses.

## METHODOLOGY

### Study Design and Participants

The present study was conducted as a pilot with a single-group pre-test–post-test design to compare the impact of a 3D-printed internal structure silicone cosmetic partial hand prosthesis on functional ability and user satisfaction against a conventional full-silicone cosmetic prosthesis.

The study population consisted of individuals with unilateral partial hand amputations from metacarpophalangeal joints to carpometacarpal joints who had previously received a conventional full-silicone cosmetic. Following approval of the research protocol by the Ethics Committee of Iran University of Medical Sciences and obtaining the ethics code (IR.IUMS.REC.1402.886), non-random convenience sampling was conducted from February to September 2024, at *Behboud Teb Prosthetics and Orthotics*, Tehran, Iran. After providing information regarding the study objectives, procedures, and required level of participation, eligible individuals who provided written consent were enrolled. Participants were not blinded due to the nature of prosthesis fabrication. During this interval, 7 individuals aged 15 to 65 years were enrolled and completed all study phases.

Inclusion criteria were: unilateral partial hand amputation at the metacarpophalangeal to carpometacarpal level, age between 15 and 65 years, prior use of a conventional full-silicone cosmetic prosthesis for at least two months (mean duration: 11.1 ± 4.1 months), absence of cognitive or neuromuscular impairments affecting upper limb function, and ability to provide informed consent.

Exclusion criteria included unwillingness to continue participation, development of complications such as skin ulcers or hypersensitivity limiting prosthesis use, irreparable prosthesis damage, or any condition interfering with daily activities (e.g., hospitalization).

### Study Implementation

Participants completed a baseline information questionnaire covering age, gender, education level, occupation, time elapsed since amputation, duration since receiving the first and current prosthesis, and daily prosthesis usage. Daily prosthesis wear time was self-reported by participants. After recording baseline data, the study variables were assessed and documented.

In this study, two types of silicone cosmetic prostheses were evaluated: a conventional full-silicone prosthesis and a silicone prosthesis incorporating a 3D-printed internal TPU structure. The conventional prosthesis had a full-silicone structure with a natural appearance, fabricated according to standard clinical practice in prosthetics and orthotics, including mold preparation, wax modeling, and silicone processing techniques.^5,15^ The TPU-based prosthesis consisted of an external layer made from medical-grade room-temperature vulcanizing (RTV) silicone (Silbione^®^, Bluestar Silicones, France; Shore A 35) combined with an internal structure designed using CAD/CAM technology extending from the residual limb end to the fingertips. Participants first completed the questionnaires based on their conventional prosthesis, including rating satisfaction with stiffness on a 0–10 numerical scale (0 = no satisfaction, 10 = complete satisfaction). They were then provided with the TPU-based prosthesis, and after four weeks of use, the same assessments were repeated.

### Outcome Measures

#### Satisfaction

User satisfaction across various aspects of the cosmetic prosthesis, including functional, aesthetic, and weight satisfaction, was assessed using the Persian version of the Trinity Amputation and Prosthesis Experiences Scales (TAPES) by Mazaheri et al.^21^ This questionnaire comprises three main sections: psychosocial adjustment, functional limitations, and satisfaction, each with three subsections. The psychosocial adjustment section includes 15 questions covering general adjustment, social adjustment, and adjustment to limitations (score range per subsection: 5–25). The functional limitations section has 12 questions addressing functional, social, and athletic limitations (score range per subsection: 3–12). The satisfaction section includes 10 questions on functional satisfaction (5 questions), aesthetic satisfaction (4 questions), and prosthesis weight satisfaction (1 question) (overall score: 10–50). The fourth section pertains to phantom pain and residual limb pain (score range each: 0–5).^22,23^

#### Satisfaction with Prosthesis Stiffness

Participants reported their satisfaction with prosthesis stiffness on a numerical scale from 0 to 10, where 0 indicated no satisfaction and 10 indicated complete satisfaction.

#### Functional Ability

Functional ability was evaluated using the Persian version of the Disabilities of the Arm, Shoulder and Hand (DASH) questionnaire. This questionnaire consists of 30 questions assessing upper limb function in daily activities on a 5-point Likert scale. The overall score ranges from 0 to 100, with higher scores indicating greater disability. A minimum of 27 responses is required for scoring. The questionnaire evaluates difficulty in performing activities, symptom severity (pain, tingling, weakness, and stiffness), and impact on social activities and body image.^24^ Translated into Persian by Mousavi et al. in 2008, its reliability and validity (ICC = 0.82) have been reported.^25^

### Mechanical Testing

#### Prosthesis Stiffness

In this study, prosthesis stiffness was assessed by measuring the maximum force applied for equivalent displacement of the fingers. Accordingly, the force-displacement curve up to the end range of motion for fingers two through five was obtained using a hydraulic materials testing device (DARTEC HA100, with 50 KN maximum load, 0.1 m/s maximum speed, 100 mm travel). The prosthesis registering higher force was considered to have greater stiffness, thereby providing a more suitable support surface.

#### Prosthesis Fatigue

Material fatigue refers to the progressive structural damage that occurs under repeated loading cycles below the ultimate strength.^26^ In this study, prosthesis fatigue limit was defined as the number of passive extension cycles of the silicone cosmetic prosthesis fingers before failure. For mechanical comparison purposes, two new prostheses were fabricated based on the mold of one representative participant: one conventional full-silicone cosmetic prosthesis and one silicone prosthesis incorporating a 3D-printed internal TPU structure. A displacement-controlled fatigue test was performed using a hydraulic material testing device (DARTEC HA100, with 50 kN maximum load, 0.1 m/s maximum speed, 100 mm travel). Cyclic loading was applied at a constant displacement of 25 mm until structural failure occurred. The prosthesis enduring more cycles before failure was considered to have a higher fatigue resistance. The fatigue test was conducted under controlled laboratory conditions for comparative evaluation and does not constitute formal standardized certification testing.

### Fabrication of Silicone Cosmetic Prosthesis with 3D-Printed Internal Structure

**Figure 1:**
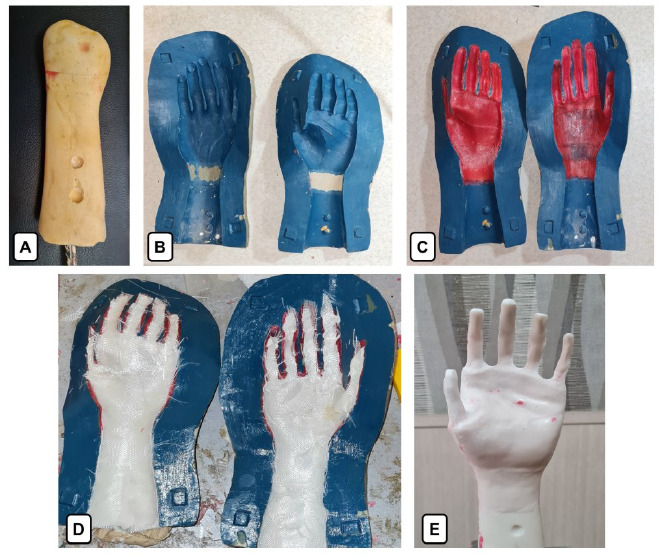
Steps of fabricating a silicone cosmetic prosthesis featuring a 3D-printed internal Structure. **A:** Positive mold derived from the patient's residual limb. **B:** Negative mold encompassing both the stump and the wax pattern. **C:** Wax layer formed to create the final silicone outer covering. **D:** Application of glass fibers and epoxy resin to establish the negative mold for the internal structure. **E:** Positive mold of the internal structure.

To fabricate this prosthesis, a negative mold of the patient's residual limb was first obtained using plaster bandages, followed by filling the mold with dental plaster. After the plaster dried, a positive mold (**Figure 1.A**) was produced, modified to match the dimensions of the remaining limb, and prepared. The amputated portion of the hand was then modeled using wax (Covex Company, Haarlem, the Netherlands) to match the dimensions of the intact side. Upon finalizing the model, a negative prosthesis mold was created from the assembly of the positive mold and mounted wax model (**Figure 1.B**). A wax layer (1-2 mm thick) was applied to the internal walls of the negative prosthesis mold to create the final silicone coating (**Figure 1.C**). To prepare the positive internal structure mold, fiberglass and epoxy resin were used to fill the remaining space in the negative prosthesis mold (**Figure 1.D**). Subsequently, the positive internal structure mold (**Figure 1.E**) and the patient's positive stump mold were scanned using a scanner (EinScan H, Shining 3D Tech Co., Ltd., China). After scanning, the internal structure design and processing were performed using Autodesk Meshmixer software (Autodesk, Inc., San Rafael, CA, USA).

### 3D Printing of the Designed Internal Structure

**Figure 2:**
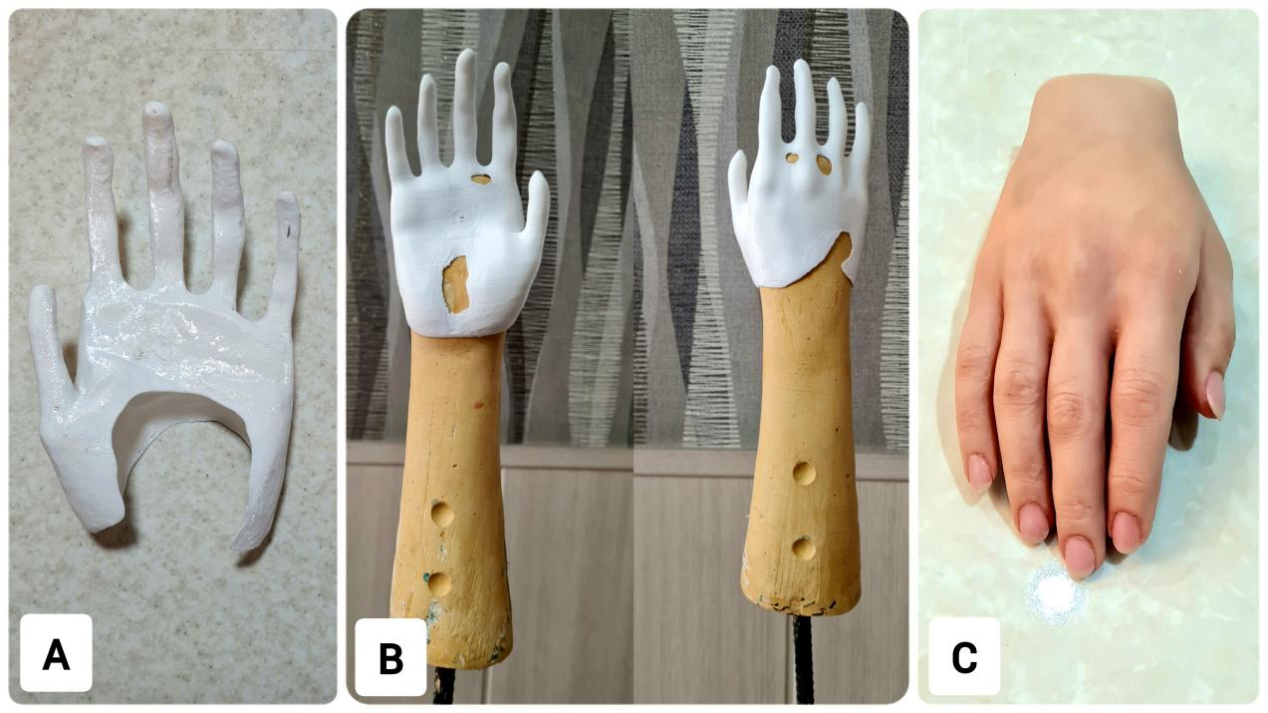
Steps of fabricating a silicone cosmetic prosthesis featuring a 3D-printed internal structure. **A:** 3D-printed internal structure. **B:** Internal structure positioned on the positive mold of patient's residual limb. **C:** Final prosthesis.

Following completion of the computer design process, the designed model was printed using the iDesign Marbin 500 3D printer (iDESIGN 3D Printing Group, Tehran, Iran) and flexible thermoplastic polyurethane filaments (Shenzhen Esun Industrial Co., Ltd., China) with 95A hardness (**Figure 2.A**). During printing, the nozzle temperature was 210°C, and the heated build plate was set to 65°C. The nozzle movement speed for printing the designed model was 40 mm/s. The internal structure wall thickness was 1.2 mm, with internal infill density at 15% of the internal structure volume. After printing the internal structure, it was placed inside the negative mold of the stump and wax model assembly (**Figure 2.B**), followed by silicone pouring. Once the silicone hardened, the silicone coating and embedded internal structure assembly were removed from the negative mold, and finishing procedures were performed. The final prosthesis fabricated by this method exhibits an appearance, color, and texture resembling a real hand, with no visual difference from the conventional full-silicone cosmetic prosthesis (**Figure 2.C**).

### Ethical Considerations

All study procedures were conducted in accordance with the Declaration of Helsinki the ethical guidelines of the Ethics Committee of Iran University of Medical Sciences. The study was approved on January 6, 2024, by the Ethics Committee of Iran University of Medical Sciences (ethics code: IR.IUMS.REC.1402.886). Informed written consent was obtained from all participants. Participation in the study was voluntary, and individuals could join or withdraw without restrictions.

### Statistical Analysis

Collected data were entered into SPSS software version 27 (Statistical Package for the Social Sciences, SPSS, Chicago, IL, USA). Data normality was assessed using the Shapiro-Wilk test. Data analysis involved paired t-tests for normally distributed variables and the nonparametric Wilcoxon test for non-normally distributed variables to compare means. In addition to significance testing, Cohen's d effect size was calculated to evaluate the clinical importance of results and provide a more precise view of effect magnitude. Cohen's d interpretation ranges are: effect size <0.2 (negligible), 0.2–0.49 (small), 0.5–0.79 (medium), and >0.8 (large).^27^ A significance level of <0.05 was applied.

## RESULTS

Seven individuals with partial hand amputation (4 males and 3 females), who had experienced amputation at least three years prior, participated in this study and completed all evaluation phases. The participants' mean age was 27.9 ± 7.6 years, with an average body weight of 67.6 ± 14.1 kg. On average, 14.7 ± 11.5 years had elapsed since their most recent amputation, and the mean time since receiving their first prosthesis was 9.1 ± 7.9 years. Additionally, the average daily use of their current (full silicone) prosthesis was 14.8 ± 2.1 hours, with a mean duration of 11.1 ± 4.1 months since acquiring this prosthesis. For the full silicone cosmetic prosthesis and the TPU prosthesis, participants reported average daily use of 8.1 ± 2.0 hours and 9.4 ± 1.1 hours, respectively.

**Table 1** presents the mean data on the mechanical and functional characteristics of the two types of silicone cosmetic prostheses (full silicone and TPU prostheses).

**Table 1:** Comparison of mean prosthesis satisfaction and functional performance in study participants (N = 7).

Variable**	Prosthesis Type	Mean ± Standard Deviation	p value	Effect Size (Cohen’s d & r***)
Functional ability (DASH)	Full silicone	53.09 ± 11.56	0.012*	1.34
	TPU	43.69 ± 11.68		
Satisfaction with prosthesis stiffness	Full silicone	4.57 ± 1.72	0.086	1.12
	TPU	7.86 ± 1.46		
Prosthesis stiffness (Newton)	Full silicone	27.29 ± 10.83	0.028*	−2.79****
	TPU	81.43 ± 27.07		
Daily usage duration (Hours)	Full silicone	8.2 ± 2.03	0.005*	−1.15
	TPU	9.43 ± 1.13		
Prosthesis final weight (kg)	Full silicone	0.29 ± 0.09	0.001*	1.98
	TPU	0.20 ± 0.05		
Prosthesis satisfaction***	Full silicone	36.00 ± 2.30	0.04*	0.76 ***
	TPU	40.57 ± 5.06		

* p < 0.05 (Significance level: 0.05)

** comparison conducted using paired t-test for normally distributed variables and Wilcoxon signed-rank test for non-normal variables.

*** Wilcoxon signed-rank test was used to compare satisfaction scores between prosthetic types (Z = −2.036, p = 0.04, r = 0.76; r = Z/√N).

**** Negative sign reflects direction of difference.

The variables include prosthesis stiffness, participant satisfaction as measured by the TAPES questionnaire, functional ability as assessed by the DASH questionnaire and prosthesis final weight results for the samples. The data indicate that the TPU prosthesis showed higher values in most measured parameters and appears to offer superior mechanical and user-related performance compared to the full silicone model. A fatigue test was performed on both prostheses of one participant. The TPU prosthesis withstood 36,500 cycles, while the full silicone prosthesis endured 13,800 cycles, showing that the TPU model had substantially greater durability and fatigue resistance.

## DISCUSSION

The present study explored the relationship between the physical characteristics of two types of cosmetic prostheses (full silicone and TPU) and both patient satisfaction and functional outcomes, particularly DASH scores. Because only seven participants were enrolled, several associations did not reach statistical significance, which calls for cautious interpretation of the results. Nevertheless, the trends that emerged provide useful direction for future work. To the best of our knowledge, no previous study has directly investigated a cosmetic partial-hand prosthesis incorporating an internal TPU core while simultaneously evaluating both mechanical properties and functional performance.

When satisfaction specifically related to prosthesis stiffness was examined, mean scores favored the TPU-based design, yet no statistically significant correlation was found between stiffness itself and overall satisfaction – a finding likely attributable to limited statistical power of the study. By contrast, the TAPES questionnaire revealed significantly higher overall satisfaction with TPU prostheses than with all-silicone devices. The large effect size associated with this difference confirms its clinical relevance and illustrates how the choice of material can meaningfully influence perceived comfort, aesthetics, and functional utility.

Analysis of DASH scores showed that individuals using TPU prostheses achieved better functional performance. Because lower DASH scores reflect less disability, these results suggest that lighter and more elastic materials such as TPU can reduce functional limitations and facilitate everyday activities. This observation is entirely consistent with earlier reports emphasizing the value of lightweight, flexible materials with appropriate mechanical behavior for improving motor control and reducing disability.^20,28,29^ Although the reduction in DASH score was statistically significant, it did not reach the commonly reported minimal clinically important difference (MCID) for the DASH questionnaire, which has been reported to range from approximately 10 to 15 points,^30^ and therefore should be interpreted with caution in terms of clinical relevance.

Daily wearing time was also significantly longer with TPU prostheses than with all-silicone ones. A potential novelty effect should be considered, as participants were aware of using a newly designed prosthesis, which may have influenced subjective outcomes such as satisfaction and wear time. The combination of lower weight and balanced stiffness appears to enhance comfort and encourage prolonged use throughout the day – a pattern that aligns closely with the higher satisfaction and better functional scores already noted. Taken together, these findings underline the importance of addressing multiple physical and functional properties simultaneously during the design process.

In terms of objective physical properties, TPU prostheses were significantly stiffer yet substantially lighter. Previous studies have reported that prosthesis weight is an important factor affecting user acceptance and comfort in upper-limb prostheses, and that increased prosthetic weight may contribute to greater muscular fatigue. Consequently, the reduced weight of the TPU prosthesis may have contributed to the increased daily wear time observed in the present study.^31,32^ In addition, previous studies have shown that TPU-based prosthetic structures can provide a favorable balance between flexibility and durability while maintaining resistance to structural failure under loading, supporting their application in prosthetic design.^33^

Ultimately, user experience with a cosmetic prosthesis emerges from a complex interplay of physical, functional, and psychological factors. Optimizing a single attribute in isolation (whether stiffness or weight) is insufficient; it is the balanced integration of weight, stiffness, ergonomic design, and appropriate mechanical response that drives meaningful improvements in satisfaction and performance – a multifactorial perspective that has been highlighted in prior research.^34,35^

In summary, prostheses incorporating TPU internal structures, thanks to their reduced weight, well-judged stiffness, and superior comfort, outperformed conventional full-silicone devices in both functional outcome and user acceptance. These findings should be interpreted cautiously given the exploratory design and potential sources of bias. Confirmation of these promising results will, however, require larger-scale studies with greater sample sizes and more comprehensive assessment tools in order to clarify the multifaceted relationships between technical design features and real-world patient experience.

### Limitations

The small sample size substantially limited the statistical power of this study, increasing the likelihood of type II errors and reducing the ability to detect true differences between prosthesis types. Additionally, due to the exploratory nature of this pilot study, no formal power analysis was conducted to determine the sample size, which may limit the statistical conclusions that can be drawn. The participant group also lacked sufficient demographic diversity in terms of age, gender, and background, restricting the generalizability of the findings to broader populations of prosthesis users. Participants were not blinded to the intervention, and the potential influence of novelty bias on subjective outcomes such as satisfaction and wear time cannot be excluded. Wear time data were self-reported and may be subject to recall bias. Furthermore, because data were collected at a single time point using a cross-sectional approach, causal relationships between prosthesis characteristics and user outcomes cannot be established, nor can long-term effects be evaluated. In addition, fatigue testing was performed on prostheses from a single participant, limiting the generalizability of mechanical durability findings.

In addition, unlike conventional prostheses incorporating metal armatures that allow manual positioning of the digits, the TPU-based internal structure used in this study does not provide this capability, which may limit grasp adaptability in certain functional tasks.

## CONCLUSION

The use of TPU as an internal structural material in upper-limb cosmetic prostheses may improve mechanical performance, increase user satisfaction, and positively influence daily quality of life. These prostheses tend to be lighter, provide a more balanced level of stiffness, and offer greater comfort during routine use, which may support individualized design approaches and encourage continued wear. Based on the findings of this study, silicone cosmetic prostheses incorporating a 3D-printed TPU core showed better functional outcomes and higher user satisfaction than conventional full-silicone models. However, the small sample size limits the extent to which these results can be generalized. Further studies involving larger cohorts and longer follow-up periods are therefore needed to confirm these findings and to better understand the wider clinical and psychosocial effects of TPU-based prosthetic designs.
